# Effect of the In Ovo Injection Site of Electrolytes on Some Biochemical Blood Parameters and Quality of Layer Chicks

**DOI:** 10.3390/ani12040532

**Published:** 2022-02-21

**Authors:** Joanna Pawłowska, Ewa Sosnówka-Czajka, Iwona Skomorucha

**Affiliations:** 1 Department of Production Systems and Environment, National Research Institute of Animal Production, 32-083 Balice, Poland; 2 Department of Poultry Breeding, National Research Institute of Animal Production, 32-083 Balice, Poland; ewa.sosnowka@iz.edu.pl (E.S.-C.); iwona.skomorucha@iz.edu.pl (I.S.)

**Keywords:** chick embryo, in ovo, quality of chicks, laying hens

## Abstract

**Simple Summary:**

In ovo technology is a unique method, the primary goal of which is to administer bioactive substances to eggs during the embryonic development of the bird. Therefore, it is necessary to develop optimal techniques for the implementation of in ovo feeding technology in practical poultry production. This preliminary study may open a window for future research on the site/location of manipulation and solutions and carriers of nutrients used for in ovo injection of laying hen embryos.

**Abstract:**

The effects of the in ovo injection site of electrolytes on selected biochemical blood parameters and the quality of layer chicks were investigated. A total of 120 fertile eggs from Rhode Island Red breeders were randomly distributed into 4 groups, with each group including 30 birds. The groups were as follows: untreated control and groups with different injection sites/locations of 500 µL of 0.9% saline (NaCl) on day 18 of incubation, i.e., into the air cell (AC), through the air cell into the amniotic fluid (AFA), and directly into the amniotic fluid (AF). Measurement at 1 day of age showed that regardless of the injection site, embryos injected with 500 µL of saline had significantly higher Tona score (95/100 points) compared to the control group (90/100 points). Chick length was similar among the injected groups (mean 14.7 cm) and shorter in the control group (13.9 cm). There was no significant effect of in ovo injection on the biochemical blood parameters: total protein, cholesterol, high-density lipoprotein, low-density lipoprotein, glucose, urea, and uric acid. The highest concentration of sodium was noted in the control group (141.59 mmol/L). Regardless of the injection site/location, chicks treated with 500 µL of NaCl were characterized by a significantly lower blood sodium concentration (by 7.45% (AC), 7.90% (AFA), and 4.84% (AF) compared with birds from the control group (*p* ≤ 0.01)). The influence of saline solution administration in ovo on the blood potassium content of chicks was demonstrated. The concentration of potassium in the control group was significantly higher (by 11.36%) than in the AC group (*p* ≤ 0.01). In conclusion, the injection of 500 µL of saline solution into the developing chick embryo during the last days of incubation may have a positive effect on the quality of day-old chicks.

## 1. Introduction

In ovo injection, although not commercially available yet, has been used for in ovo feeding of embryos [1]. Research has been conducted for many years on in ovo feeding of nutrients for potential improvement of poultry production quality. A great deal of research has been conducted over the last 25 years that has investigated the injection sites, including the commonly accepted method (US patent no. 6,592,878) proposed by Uni and Ferket [2]. This method involves the administration into the embryonic amnion of a solution or suspension of nutrients at 17–18 days of incubation, which allows the feeding solution to be swallowed with the amniotic fluid and absorbed in the intestine. In ovo feeding has triggered a large number of studies concerning the delivery of various active agents: amino acids [3], carbohydrates [4], and vitamins [5] and/or other substances. The results of these publications suggest improvements in rearing parameters (especially for day-old chicks) and fattening performance, which, from an economic point of view, translates into profitable poultry production.

It is necessary to develop optimum techniques for the implementation of in ovo feeding technology in practical poultry production as a method for supplementing avian embryos since many factors contribute significantly to the effectiveness and efficiency of the procedures. These include the injection site/location. During the later stage of egg incubation, there are several locations through which an in ovo injection may be delivered: the embryo body, the yolk sac, the amniotic/allantoic fluid, and the air cell [6]. At the same time, every in ovo manipulation disrupts the embryonic environment and nutrient in ovo injection may upset the osmotic balance, especially since this “manual” procedure requires high precision from the operator. These considerations suggest that during in ovo injection of vaccines, injection depth is crucial; the needle not being deep enough in the egg will make the vaccine less effective while injecting the needle too deep may cause trauma to the embryo [6,7,8].

The solutions and carriers of nutrients used for in ovo injection include physiological saline [9,10], deionized water [11,12], and commercial carriers of vaccines [13]. Furthermore, McGruder et al. [14] indicated that among the analyzed solutions, potassium chloride (KCl) and tripotassium citrate (C6H5K3O7) within certain concentration and volume ranges have the greatest potential for use individually or in combination for the commercial injection of hatching eggs. One of the most common agents is physiological saline (0.45–0.9%), especially since it is neutral to tissues and its osmolality is similar to that of blood. Another important aspect of the in ovo procedure is the volume of the injection solution. The most commonly used volume (e.g., of a vaccine against Marek’s disease) is 50 µL. Nevertheless, a carbohydrate solution with a volume as high as 700 µL allows 90% hatchability from fertile eggs [15]. According to McGruder et al. [14], solution volumes equal to or greater than 2000 µL should be avoided.

The role of the injection carrier and in ovo site/location is crucial as far as the effectiveness and efficiency of in ovo manipulation is concerned. However, many experimental studies regarding the in ovo method have not taken into account the carrier used for in ovo nutrient solvent (NaCl) on the metabolic status, biochemical blood parameters, and chick value of chicks. In addition, very few studies have been conducted on layer hens using in ovo technology. In this research, layer embryos were used as animal models to study the effects of the injection site of electrolytes on some biochemical blood parameters and the quality of layer chicks.

## 2. Materials and Methods

All experimental procedures were evaluated and approved by the Local Ethics Committee in Krakow (158/2018, 18 July 2018).

### 2.1. Animals and Experimental Design

The experiment used 155 hatching eggs from a flock of Rhode Island Red hens aged 36 weeks. Eggs were individually weighed, and egg mass averaged 58.9 g. Eggs were incubated according to standard hatchery practices (T = 37.7 °C, relative humidity (RH) = 55%) and turned automatically every 2 h in an incubator Mini IV (Fest, Gostyń, Poland).

On day 17.5 of incubation, eggs were candled to remove those that were unfertilized or contained dead embryos. A total of 120 eggs were randomly divided into 4 treatment groups, with each group containing 30 eggs, and were equally represented on each of the 3 replicate trays. The experimental groups were the non-injected control group (Control), and three groups with different injection sites/locations, i.e., into the air cell (AC), through the air cell into the amniotic fluid (AFA) (methodology proposed by Uni et al. [2], and through the eggshell directly into the amniotic fluid (AF) (Table 1). The last 3 groups were manually injected with 500 µL of 0.9% NaCl (Glenmark, Warsaw, Poland). The optimum volume of solution was recommended by Kalantar et al. [16]. Before the manipulation, the injection site of the eggshell was sterilized with a solution of 70% ethyl alcohol. All injected solutions were prepared on the day of injection. Solutions were heated to 37 °C in an incubator prior to the in ovo injection procedure. The injection was performed through the hole in the shell using a 21G needle. After injection, the window was sealed with Parafilm^®^, and the eggs were transferred into 4 sanitized hatching baskets (T = 36.5 °C, RH = 60–65%).

### 2.2. Hatchability and Quality of Chicks

Upon hatching, the live hatched chicks were weighed individually on an electric balance. Hatchability was calculated as the number of hatched chicks as a percentage of the injected eggs. The quality of newly hatched chicks was scored from the external appearance of the birds on a 100-point scale according to Tona et al. [17]. Based on this score, the quality of each chick was evaluated based on eight criteria: (1) activity; (2) down and appearance; (3) retracted yolk; (4) eyes; (5) legs; (6) navel; (7) remaining membrane; and (8) remaining yolk. The quality score was calculated by summing up the scores for these characteristics. Chick length was measured from the tip of the beak to the end of the middle toe [18].

### 2.3. Sample Collection and Laboratory Analyses

Chicks’ blood was sampled from 9 randomly chosen chicks from each experimental group (3 birds per replicate) to determine total protein (B6528-125), cholesterol (C6608-100), high-density lipoprotein (H6421-080), low-density lipoprotein (L6410-080), glucose (G6620-100), urea (M6652-075), uric acid (K6681-100), and electrolytes: sodium and potassium (4102). Blood was collected on day 1 of age by decapitation. The collected blood was centrifuged for 10 min at 3000 rpm (MPW-52, MPW Med. Instruments, Warsaw, Poland) and the plasma separated by centrifugation was transferred to Eppendorf tubes (1.5 mL). The biochemical parameters and plasma Na and K levels were analyzed with a Mindray BS-120 biochemistry analyzer using Alpha Diagnostics (Warsaw, Poland) reagent kits and procedures.

### 2.4. Statistical Analysis

Based on the results obtained, the mean values and standard error of the mean were calculated for each trait, tabulated, and presented in the Results section. In addition, the data were statistically analyzed using analysis of variance (ANOVA), and significant differences between the means were determined with Duncan’s multiple range test. The statistical analyses were performed using Statistica software (version 13.0, TIBCO Software Inc., Palo Alto, CA, USA). All *p* < 0.05 were considered significant.

## 3. Results

### 3.1. Hatchability and Chick Quality

The hatchability percentage of injected eggs showed no significant difference between groups (*p* > 0.05) (Table 2).

Bird quality was assessed based on the following parameters: body weight, chick length, and Tona score (Table 2). Treatment factors had no significant effect on chick weight (*p* > 0.05). Regardless of the injection site/location, embryos injected with 500 µL of NaCl showed a significantly higher Tona score (95/100) compared to control embryos (90/100). These differences were statistically significant at *p* < 0.05. Chick length values were similar in the injected groups (14.7 cm on average) and lowest in the control group (13.9 cm). The differences were significant at *p* < 0.001. When analyzing all traits together, the lowest quality was noted in the control chicks.

### 3.2. Biochemical Blood Parameters

The mean levels of selected biochemical parameters in the plasma of newly hatched chicks are shown in Table 3. This test showed that the injection of physiological saline into the developing embryo did not have a significant effect on the plasma levels of biochemical parameters in the chicks.

Table 4 presents the mean concentration of electrolytes in the plasma of newly hatched chicks. Significant differences were observed for sodium content, the highest level of which was found in the control group (141.59 mmol/L). Regardless of the injection site/location, chicks injected with 500 µL of NaCl were characterized by a significantly lower concentration of sodium (by 7.45% in AC, by 7.90% in AFA, and by 4.84% in AF) compared to the control birds (*p* < 0.01). A similar situation occurred for the potassium concentration in the blood of newly hatched chicks, where the treatment factor had a differentiating effect. In ovo administration of physiological saline solution had an effect on the blood potassium content in the chicks. This element was significantly higher (by 11.36%) in the control group compared to the AC group (*p* < 0.01).

## 4. Discussion

Neither in ovo injection of physiological saline on day 18 of incubation nor the injection site showed a significant difference in hatchability among the groups. This is consistent with Kop Bozbay et al. [19], who showed no significant differences in the level of hatchability for NaCl injection into the air cell or amnion of embryos. Similar hatchability results were obtained by Moghaddam et al. [20], who injected physiological saline solution into the air cell and yolk sac of the embryo. Studies conducted to date, including the present one, suggest that in ovo manipulation does not improve chick hatchability. According to Uni and Ferket [21], the proposed maximum osmolality of a feeding solution for in ovo injection ranges from 500 to 600 mOsm/L for optimum hatchability. Therefore, it can be supposed that the applied 0.9% NaCl with osmolality of 308 mOsm/L did not significantly influence the hatchability between the groups.

With regard to economic aspects, the major goal of a hatchery is to obtain a high hatchability percentage while the production results are determined by chick quality and health. There is no question that chick quality is made up of many parameters, the objective evaluation of which may help choose chicks with the highest production potential [22]. Data from the literature suggest that in ovo injection may improve the weight of chicks at hatching, especially when carbohydrates are used [10,11,23]. Some researchers also highlighted that the particular solvent used for in ovo injection may affect embryo growth [14]. A recent study reported no changes in the body weight of newly hatched chicks that had been injected with physiological saline [24]. These results are in agreement with ours, regardless of the injection site/location. Although the body weight of newly hatched birds is often used as a benchmark for quality, the authors of numerous publications do not agree on its relationship with the final slaughter weight [25,26]. The technique for scoring chick quality elaborated by Tona et al. [17] remains one of the most common methods used in research and in practical breeding to assess the condition of day-old chicks. This 100-point scale was originally developed to assess the quality of day-old broiler chicks [17]. It is worth noting that in our study, the highest scores among the study groups were awarded to all the injected chicks. Watanabe et al. [27], who injected a physiological saline solution (100 µL of 0.9% NaCl) into the air cell of control birds on day 18 of incubation, observed higher Tona scores in the experimental groups treated with corticotropin-releasing hormone. Another reliable parameter is chick length at hatching, measured from the tip of the beak to the end of the middle toe [28]. In our study, by analogy with the Tona score, we noted a favorable effect of the physiological saline on the chick length in the injected groups. The difference between the experimental and control groups exceeded 0.8 cm on average. These results correlated with the results of studies by Salahi et al. [18] conducted on broiler chicks. The authors demonstrated that the injection of normal saline (NaCl 0.9%) into the amnion at 445 and 453 h of incubation positively influenced the chick length (0.4 and 0.2 cm more than control group, respectively). The effect of saline injection on the chick length has not been clear and it is difficult to explain. One can suppose that the improvement of the chick length was related to utilization of the available yolk. Some authors have reported that hatchling length is related to yolk-free body mass and predicts subsequent performance [29]. As regards our research, when analyzing the above traits together, it is concluded that the lowest quality of chicks was noted in the control group (non-injected).

The hatching egg content is a mineral component for the developing embryo and depends on the location in the egg. The main location of sodium and potassium is in the egg albumen. During incubation, the albumen content is integrated with the yolk sac and the amniotic fluid, enabling the embryo to consume Na and K [30,31]. This process starts around 13 days of hatching and continues until internal pipping on day 19 of incubation [32]. Sodium and potassium level imbalances may cause energy wastage due to excessive ion transfer within hatching eggs. In turn, trace mineral deficiencies may cause impaired growth, abnormal development of the organs, and, in extreme deficiencies, death of the embryo [33]. In our study, all the injected embryos had higher blood sodium levels on the hatching day compared to the control group, where the concentration of this element was the lowest. A similar situation occurred for the potassium concentration in the blood of newly hatched chicks, where the treatment factor had a differentiating effect. McGruder et al. [14] noted that the introduction of external electrolytes into the developing embryo environment may disrupt the normal concentration gradients necessary for the influx of water to embryonic tissues. Despite this, we detected no differences between the groups in the hatchability.

## 5. Conclusions

In summary, it is concluded that the in ovo injection site/location (into the air cell, through the air cell into the amniotic fluid, and directly into the amniotic fluid) showed no significant differences regarding the hatchability percentage, quality of chicks, and biochemical blood parameters of Rhode Island Red chicks, which indicates that the injection manipulation and injection carrier are safe for the embryos. Furthermore, the present results suggest that the injection of 500 µL of carrier itself (physiological saline) into the developing chick embryo during the last days of incubation may have a positive effect on the quality of day-old chicks. Moreover, the injection of saline may affect the sodium and potassium levels in the plasma of newly hatched chicks.

## Figures and Tables

**Table 1:** Groups of eggs for in ovo injection.

Treatment Group	In Ovo Injection
Control	Non-injected
AC	0.5% NaCl into the air cell
AFA	0.5% NaCl through the air cell into the amniotic fluid
AF	0.5% NaCl through the eggshell directly into amniotic fluid

**Table 2:** Effect of in ovo injection of saline on the hatch results and quality of one-day-old layer chicks.

Item	Injection Sites				SEM	*p*-Value
	Control	AC	AFA	AF		
Hatchability (%)	79.32 ± 0.58	76.59 ± 0.55	76.65 ± 0.56	78.56 ± 0.59	0.43	0.061
Chick weight (g)	41.61 ± 0.56	42.33 ± 0.68	42.38 ± 0.66	42.91 ± 0.65	0.32	0.556
Tona score	90.17 ± 1.61 ^b^	94.35 ± 1.39 ^a^	94.56 ± 1.39 ^a^	94.86 ± 1.02 ^a^	0.71	0.044
Chick length (cm)	13.92 ± 0.10 ^b^	14.92 ± 0.15 ^a^	14.57 ± 0.12 ^a^	14.70 ± 0.10 ^a^	0.07	<0.001

^a,b^—values within a row with different letters differ significantly (*p* ≤ 0.05). Control—non-injected; AC—injected into the air cell; AFA—injected through the air cell into the amniotic fluid; AF—injected into the amniotic fluid. *n* = 3; 8 observations were pooled within each replicated tray; values are presented as means ± SE.

**Table 3:** Effect of in ovo injection of saline on total protein (TP), cholesterol (Chol), high-density lipoprotein (HDL), low-density lipoprotein (LDL), glucose (GLU), urea (U), and uric acid (UAc) of newly hatched layer chicks.

Item	Injection Sites				SEM	*p*-Value
	Control	AC	AFA	AF		
TP (g/L)	21.5 ± 1.94	20.3 ± 0.62	22.5 ± 1.11	20.0 ± 0.72	0.605	0.460
Chol (mmol/L)	13.50 ± 1.16	14.34 ± 1.09	14.53 ± 1.23	13.28 ± 0.77	0.526	0.808
HDL (mmol/L)	4.20 ± 0.20	4.77 ± 0.84	4.25 ± 0.20	4.07 ± 0.34	0.231	0.735
LDL (mmol/L)	4.94 ± 0.37	6.04 ± 0.53	6.41 ± 0.51	4.82 ± 0.53	0.260	0.065
GLU (mmol/L)	14.47 ± 0.75	13.64 ± 0.24	13.76 ± 0.68	12.85 ± 0.63	0.308	0.332
U (mmol/L)	4.40 ± 0.16	4.17 ± 0.19	4.83 ± 0.25	4.48 ± 0.25	0.111	0.209
UAc (µmol/L)	294.23 ± 34.4	247.84 ± 19.4	287.08 ± 42.0	299.4 ± 45.7	17.968	0.752

Control—non-injected; AC—injected into the air cell; AFA—injected through the air cell into the amniotic fluid; AF—injected into the amniotic fluid. *n* = 3; 3 observations were pooled within each replicated tray; values are presented as means ± SE.

**Table 4:** Effect of in ovo injection of saline on macroelements’ concentrations in the blood of newly hatched layer chicks.

Item	Injection Sites				SEM	*p*-Value
	Control	AC	AFA	AF		
Na (mmol/L)	141.59 ± 1.29 ^a^	131.04 ± 2.07 ^b^	130.49 ± 1.74 ^b^	134.73 ± 2.05 ^b^	1.12	<0.01
K (mmol/L)	5.81 ± 0.16 ^a^	5.15 ± 0.14 ^b^	5.43 ± 0.14 ^b^	5.56 ± 0.13 ^b^	0.08	0.013

^a,b^—values within a row with different letters differ significantly (*p* ≤ 0.05); Control—non-injected; AC—injected into the air cell; AFA—injected through the air cell into the amniotic fluid; AF—injected into the amniotic fluid. *n* = 3; 3 observations were pooled within each replicated tray; values are presented as means ± SE.

## Data Availability

All data are available from the authors’ database.
